# Polycystic liver disease presenting with an exudative pleural effusion: a case report

**DOI:** 10.1186/1752-1947-6-107

**Published:** 2012-04-13

**Authors:** Kerry Woolnough, Altav Palejwala, Simon Bramall

**Affiliations:** 1 Department of Gastroenterology, Queen's Hospital, Burton-on-Trent, West Midlands, UK; 2 University Hospital of Birmingham, West Midlands, UK

## Abstract

**Introduction:**

Polycystic liver disease is asymptomatic in 95% of patients. In the remaining 5% it causes symptoms due to the local mass effect of the polycystic liver. We describe the case of a patient who presented with symptoms of a pleural effusion and was also found to have polycystic liver disease. The effusion recurred despite repeated efforts at drainage and only resolved following surgical debridement of the cystic liver.

**Case presentation:**

A 50-year-old Caucasian woman presented with a two-week history of increasing dyspnoea. An examination revealed a large right pleural effusion and gross hepatomegaly. An ultrasound confirmed a large polycystic liver and diagnostic thoracocentesis revealed an exudate, which was sterile to culture. The pleural effusion proved refractory to drainage and our patient underwent surgery to deroof the main hepatic cysts in an attempt to reduce the pressure on her right diaphragm. The histology was compatible with that of polycystic liver disease. No evidence of malignancy was found. After surgery, our patient had no recurrence of her effusion and, to date, has remained asymptomatic from her polycystic liver disease.

**Conclusion:**

The case in this report illustrates that an exudative pleural effusion is a rare complication of polycystic liver disease. We feel that the mechanical effects of a large polycystic liver, and subsequent disruption of sub-diaphragmatic capillaries, resulted in a persistent exudative pleural effusion. Thus, surgical debulking of the hepatic cysts is required to manage these effusions.

## Introduction

Polycystic liver disease is a relatively benign condition, causing significant symptoms in less than 5% of patients. The most common presentations necessitating surgical intervention are due to the mass effect of cysts within the liver, causing abdominal pain and distension, obstructive jaundice, portal hypertension and recurrent ascites [1-4]. There have been several reports in the literature describing the complications of polycystic liver disease [5]; however, presentation with a recurrent exudative pleural effusion, only resolving after debridement of hepatic cysts, has rarely been described. We feel that this should be considered as a recognised complication of polycystic liver disease and also be an indication for surgical intervention.

## Case presentation

A 50-year-old Caucasian woman presented with increasing dyspnoea over a two-week period and a two-month history of right upper quadrant distension and discomfort. She did not smoke and denied any systemic symptoms of fever, night sweats, weight loss, arthralgia, arthritis or skin rashes. No cardiac, neurological or other gastrointestinal symptoms were reported.

She had no past medical history of note and no previous exposure to asbestos, animals or the farming community. She had no history of trauma or recent travel. She did not report any family history of polycystic kidney or liver disease. She did not drink any alcohol.

**Figure 1:**
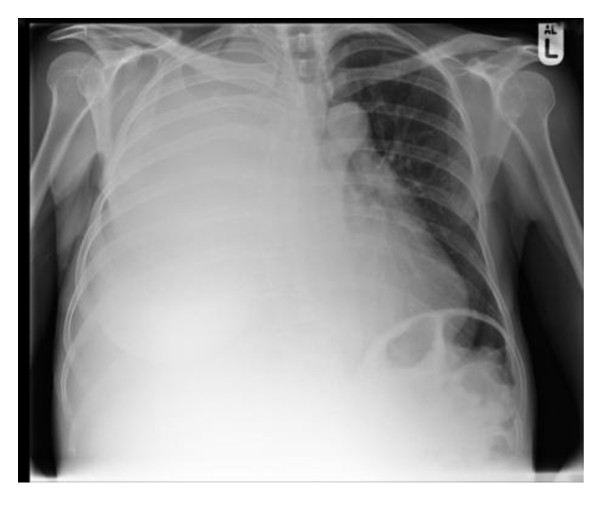
**X-ray of patient's chest revealing a right-sided pleural effusion**.

An examination revealed a large right pleural effusion, confirmed on X-ray of her chest (Figure 1), and gross hepatomegaly. No other abnormalities were detected. In particular, there was no lymphadenopathy or any evidence of cardiac compromise or decompensated chronic liver disease.

Laboratory investigations revealed that she had normal renal function with an estimated glomerular filtration rate > 60 mL/min. Her liver function tests showed a slightly raised alkaline phosphatase level of 145 U/L and a slightly low albumin of 29 g/L. Her levels of total bilirubin (12 μmol/L), alanine transaminase (9 U/L), total protein (57 g/L) and globulin (28 g/L) were all normal. A full blood count demonstrated a slightly raised platelet count of 474 × 10^9^/L. Her haemoglobin, white cell count differentials and coagulation screen were all entirely normal. A serum hydatid enzyme-linked immunosorbent assay was negative.

**Figure 2:**
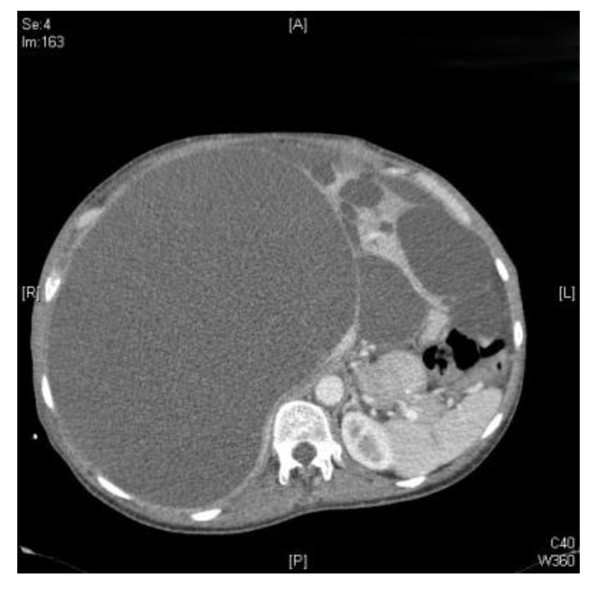
**A polycystic liver with a single large cyst (arrow) measuring 20 × 21 × 18 cm**.

Contrast enhanced computer tomography (CT) of her chest, abdomen and pelvis confirmed a large right pleural effusion, with a shift of the mediastinum to the left and a raised right hemidiaphragm. No cystic lesions were identified within the thoracic cavity. There was no evidence of a bronchopulmonary or bronchobiliary fistulae or pulmonary embolism. A polycystic liver with a large cyst measuring 20 × 21 × 18 cm (Figure 2) was found to be displacing the right hemidiaphragm superiorly and her upper abdominal structures posteromedially.

Two pleural aspirates were performed on separate occasions and confirmed the effusion as an exudate (with protein values of 47 and 38 g/L). The pleural fluid pH (8.0), glucose (6.1 mmol/L), lactate dehydrogenase (321 U/L) and amylase (16 U/L) did not indicate the cause to be due to infection, pancreatitis or an autoimmune condition. Microscopy revealed no organisms, acid fast bacilli, ova, cysts or parasites. The pleural fluid remained sterile to culture for fungi, mycobacterium and bacteria. No malignant cells were seen on cytology.

Our patient was admitted on several occasions for repeated thoracocentesis. No cause was found for the persistent exudative pleural effusion and, after discussion at the hepatobiliary multidisciplinary meeting, it was felt that the large right hepatic cyst pushing on her right hemidiaphragm was the most likely explanation for this patient's recurrent problems. Deroofing of the large hepatic cyst was advised to improve both her abdominal symptoms and to also prevent the recurrence of her pleural effusion.

Our patient underwent surgery two months after presentation to deroof the main hepatic cyst. The histology of the liver cyst, retrieved during surgery, demonstrated the cyst wall to be composed of dense fibrous tissue, with the inner aspects containing areas of haemorrhage, ulceration and granulation tissue. The outer aspects showed small nodules of entrapped hepatocytes together with a few scattered bile ducts. No epithelial lining or any evidence of malignancy was identified. This was felt to be compatible with that of polycystic liver disease. No evidence of hydatid disease was found.

After surgery, our patient has had no recurrence of her effusion and has remained asymptomatic from her polycystic liver disease.

## Discussion

Exudative pleural effusions develop when there is a change in the permeability of local capillaries or the pleural surface. Common causes include malignancy and parapneumonic effusions. Less common triggers include pulmonary infarction, rheumatoid arthritis, autoimmune disorders, asbestosis and pancreatitis. Rarely, yellow nail syndrome, drugs and fungal infections lead to exudative effusions [6]. In this patient, no evidence of malignancy, infection or infarction was found. She was on no regular medication, had no history of asbestos exposure nor any features suggestive of an autoimmune process. Analysis of the pleural fluid aspirate made the possibility of tuberculosis, a connective tissue disorder or pancreatitis very unlikely. Hydatid disease was felt to be one possibility. However, analysis of pleural fluid, cyst fluid, liver cyst histology and a negative serum enzyme-linked immunosorbent assay excluded this. No communication between the peritoneal and pleural cavity was found on CT, excluding a fistula and/or diaphragmatic defect, or rupture of a liver and/or hydatid cyst, all of which have been reported as mechanisms for pleural effusions associated with a polycystic liver [1,7,8].

We therefore postulate that the recurrent right-sided pleural effusion occurred as a direct consequence of the mass effect of the main hepatic cyst displacing and deforming the right hemidiaphragm. This in turn led to a disruption of the local capillary permeability and pleural inflammation, resulting in a persistent exudative effusion.

Pleural effusions have rarely been reported as a complication of polycystic liver disease. We found one case in the literature of a symptomatic large right-sided pleural effusion complicating polycystic liver disease and requiring intervention, reported by van Erpecum *et al.*, although this was also associated with ascites and attributed to a 'abdomino-pleural communication' [4].

The pathogenesis of a recurrent, right-sided exudative pleural effusion in association with polycystic liver disease remains unclear, with no mechanism described in the literature. As the pleural effusion did not recur after the main hepatic cyst was removed, we felt that the mass effect of the hepatic cyst, disrupting the diaphragmatic surface and causing inflammation of the parietal pleura, to be the explanation for this patient's recurrent exudative effusion.

Polycystic liver disease has a variable natural history, with the majority of patients seen to remain asymptomatic with normal liver function tests [1,3]. In a retrospective case series review of 53 patients with polycystic liver disease, 45.1% of patients had hepatomegaly, 36.5% reported pain, 9.6% dyspnoea and 9.6% reduced mobility. Complications requiring intervention in this series of patients were cyst bleeding in 12.5%, cyst rupture in 12.5% and cyst infection in 30%. Portal hypertension was developed by 2.5% of the patients and 5% received a liver transplant [1]. Biliary and inferior vena cava obstruction, chronic abdominal pain and symptomatic abdominal distension are also criteria to offer surgical intervention [2].

Symptomatic polycystic liver disease has a known female preponderance and is linked to multiparity. In addition, the size of cysts increases with exposure to elevated oestrogen levels in pregnancy and with the use of exogenous female steroids [6,9]. The medical management involves cimetidine to reduce secretin production in unroofed cysts [9] and the avoidance of hormonal therapy [6]. It is frequently infective. Thus, surgery remains the mainstay of treatment when patients become symptomatic. Surgical intervention comprises cyst aspiration and sclerosis, fenestration with and without hepatic resection and orthotopic liver transplantation [5].

## Conclusion

The abdominal complications caused by the pressure effect of large cysts that develop from polycystic liver disease are well described. Thoracic complications, such as pleural effusions, are not. This case demonstrates that the size of the cysts and the pressure they cause on adjacent structures (in this case the diaphragm) are frequently the cause of complications in polycystic liver disease, here causing a recurrent pleural effusion.

As our patient's effusion resolved after surgical intervention, we are of the opinion that all patients presenting in this way should be referred to a hepatobiliary centre for the consideration of operative management.

## Consent

Written informed consent was obtained from the patient for publication of this case report and any accompanying images. A copy of the written consent is available for review by the Editor-in-Chief of this journal.

## Competing interests

The authors declare that they have no competing interests.

## Authors' contributions

AP and SB analysed and interpreted the patient data regarding polycystic liver disease and its association with the presenting pleural effusion. SB performed the surgical debridement of the hepatic cyst. KW was involved in the inpatient care and was the major contributor in writing the manuscript. All authors read and approved the final manuscript.

## Authors' information

SB is a consultant hepatobiliary surgeon at the University Hospital of Birmingham.

## References

[B1] Bistritz L Tamboli C Bigam D Bain VG Polycystic liver disease: experience at a teaching hospital Am J Gastroenterol 2005 100 10 2212 2217 10.1111/j.1572-0241.2005.50258.x 16181371

[B2] Turnage RH Eckhauser FE Knol JA Thompson Therapeutic dilemmas in patients with symptomatic polycystic liver disease Am Surg 1988 54 6 365 372 3288024

[B3] Russell RT Pinson CW Surgical management of polycystic liver disease World J Gastroenterol 2007 13 38 5052 5059 17876869 10.3748/wjg.v13.i38.5052 PMC4434633

[B4] Van Erpecum KJ Janssens AR Terpstra JL Tjon A Tham RJ Highly symptomatic adult polycystic disease of the liver. A report of fifteen cases J Hepatol 1987 5 1 109 117 10.1016/S0168-8278(87)80068-5 3655307

[B5] Arnold HL Harrisson SA New advances in evaluation and management of patients with polycystic liver disease Am J Gastroenterol 2005 100 11 2569 2582 10.1111/j.1572-0241.2005.00263.x 16279915

[B6] Karimberg AA Loffeld Multiple cysts in the liver autosomal dominant polycystic liver disease Neth J Med 2006 64 6 199 201 16788219

[B7] Bird R Fagen K Silverman ED A case of bronchobiliary fistula in the setting of adult polycystic kidney and liver disease, with a review of the literature Clin Nucl Med 2005 30 5 326 328 10.1097/01.rlu.0000159674.33299.eb 15827402

[B8] Lane RA Colapinto ND Biliobronchial fistula following pyogenic liver abscess Can J Surg 1983 26 6 521 522 6627143

[B9] Newman KD Torres VE Rakela J Nagorney DM Treatment of highly symptomatic polycystic liver disease Ann Surg 1990 212 1 30 37 10.1097/00000658-199007000-00005 2363601 PMC1358071

